# Multimodality Imaging of Breast Parenchymal Density and Correlation with Risk Assessment

**DOI:** 10.1007/s12609-019-0302-6

**Published:** 2019-01-17

**Authors:** Georg J. Wengert, Thomas H. Helbich, Doris Leithner, Elizabeth A. Morris, Pascal A. T. Baltzer, Katja Pinker

**Affiliations:** 1 Department of Biomedical Imaging and Image-guided Therapy, Division of Molecular and Gender Imaging, Medical University of Vienna, Vienna, Austria; 2 Department of Diagnostic and Interventional Radiology, University Hospital Frankfurt, Frankfurt, Germany; 3 Department of Radiology, Breast Imaging Service, Memorial Sloan Kettering Cancer Center, 300 E 66th St, 7th Floor, New York, NY 10065, USA

**Keywords:** Breast density, Mammography, Ultrasound, Magnetic resonance imaging, Fibroglandular tissue, Breast cancer

## Abstract

**Purpose of Review:**

Breast density, or the amount of fibroglandular tissue in the breast, has become a recognized and independent marker for breast cancer risk. Public awareness of breast density as a possible risk factor for breast cancer has resulted in legislation for risk stratification purposes in many US states. This review will provide a comprehensive overview of the currently available imaging modalities for qualitative and quantitative breast density assessment and the current evidence on breast density and breast cancer risk assessment.

**Recent Findings:**

To date, breast density assessment is mainly performed with mammography and to some extent with magnetic resonance imaging. Data indicate that computerized, quantitative techniques in comparison with subjective visual estimations are characterized by higher reproducibility and robustness.

**Summary:**

Breast density reduces the sensitivity of mammography due to a masking effect and is also a recognized independent risk factor for breast cancer. Standardized breast density assessment using automated volumetric quantitative methods has the potential to be used for risk prediction and stratification and in determining the best screening plan for each woman.

## Introduction

Breast density, or breast composition, indicates the amount of fibroglandular tissue in relation to fatty tissue within the breast. Breast tissue composition exists in high variability among women, and is also subject to change during life, influenced by endogenous (age, parity, body mass index, ethnicity) and exogenous (smoking, alcohol, obesity, sedentary lifestyle, oral contraception, hormone replacement therapy) factors (Table 1) [1–4]. Breast density impacts the risk for breast cancer in different ways. There is a strong body of evidence that the sensitivity of mammography for breast cancer detection decreases with increasing breast density [5, 6]. This so-called masking effect is due to an overlap with normal breast tissue thus obscuring breast lesions and is most pronounced in extremely dense breast parenchyma [7, 8]. In addition to causing false-negative findings due to the low efficiency of screening mammography [9], breast density may also lead to increased false-positive and recall rates [10, 11].

Although the masking effect of breast cancer in dense breast is important, it must be noted that the association between breast density and risk for breast cancer is not merely a masking bias and cannot be explained by the reduced sensitivity of mammography alone. Several studies have consistently shown that breast density is also an independent and strong risk factor for breast cancer [12–17]. The consistent association between increased density and cancer risk emphasizes its potential for risk prediction and risk stratification, and this might become a valuable tool in determining the best screening plan for each woman.

In the past decade, breast density notification laws have been passed with the intent to inform women about their own breast density and possible benefits from supplemental screening methods, such as breast ultrasound. As of April 2018, 35 states in the USA have mandated that patients and her primary care physicians be notified with patients’ breast density status and the risk posed by the breast density [18•]. Breast density legislation provides a unique opportunity to strengthen patient-provider relationships by encouraging physicians to engage women in a conversation about the limitations, risks, and benefits of screening, as well as to provide women with greater autonomy.

This review will provide a comprehensive overview of the currently available modalities for qualitative and quantitative breast density assessment and the current evidence on breast density and breast cancer risk assessment.

## Breast Density Assessment

### Mammography

The assessment of breast density is usually performed on the mammogram and is defined as the amount of fibroglandular tissue relative to the fatty tissue. On mammography, the fatty components appear radiolucent, while the fibroglandular components, consisting of epithelial and stromal tissue, appear radiopaque. The mammogram is a 2D image as a 3D structure. There are several methods available for assessing the mammographic density, ranging from subjective visual assessment to semi-automated methods and automated methods. Currently, there are no recommendations or criteria for standardized breast density assessment [19, 20]. The subjective or pattern-based assessment methods include Wolfe’s classification [21], Tabar patterns [22], Boyd, and Breast Imaging Reporting and Data System (BI-RADS) of the American College of Radiology (ACR) [6]. Semi-automated methods include Cumulus and Madena. Fully automated area-based mammographic density assessment methods include ImageJ, AutoDensity, LIBRA, STRATUS, MedDensity, iReveal, and Densitas. Fully automated volumetric-based methods are BD_SXA_, Cumulus V, Quantra, and Volpara Density.

**Fig. 1:**
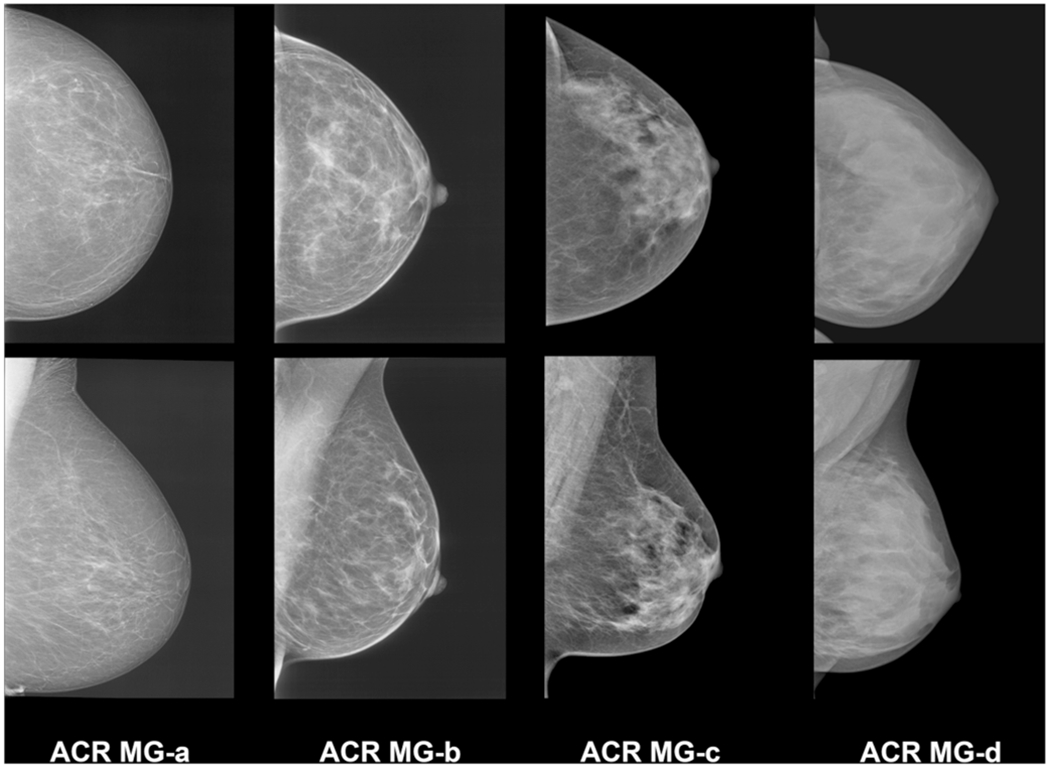
Example images of the four breast density/composition categories defined by the 5th edition of the BI-RADS mammography atlas with descriptive categories indicating coalescent breast tissue with possible masking of underlying masses. ACR MG-a, the breasts are almost entirely fatty; ACR MG-b, there are scattered areas of fibroglandular density; ACR MG-c, the breasts are heterogeneously dense, which may obscure small masses; and ACR MG-d, the breasts are extremely dense, which lowers the sensitivity of mammography

The BI-RADS lexicon is the most widely used method clinically by radiologists. The current revised 5th edition of the BI-RADS atlas defines the four breast density/composition categories as follows: ACR-MG-a, the breasts are almost entirely fatty; ACR-MG-b, there are scattered areas of fibroglandular density; ACR-MG-c, the breasts are heterogeneously dense, which may obscure small masses; and ACR-MG-d, the breasts are extremely dense, which lowers the sensitivity of mammography [6]. Women classified as either ACR-MG-a or ACR-MG-b are considered as having non-dense breasts, whereas women with ACR-MG-c or ACR-MG-d are considered as having dense breasts. Moreover, there has been a change from the previous edition [23] to the revised 5th edition of the BI-RADS atlas, from a percentage categorization of total breast density with quartiles (< 25%; 25–49%; 50–74%; and > 75%) to descriptive categories and coalescent areas on the mammogram to indicate possible masking of underlying breast masses (Fig. 1). This masking effect is one of the primary reasons for the decreased sensitivity of mammography in higher density categories introducing a potential benefit of supplemental screening [24•]. Many studies have shown significant inter- and intra-observer variability for subjective visual estimation of breast density/composition on mammography [25–27, 28••], which implies that qualitative assessment is a rather imperfect measure of breast density [29••].

To overcome the limitations of subjective visual assessments, computer-aided semi-automated and fully automated measurement approaches are now available and can be either a 2D area-based or 3D volumetric-based methods for assessing breast density. The most commonly used 2D technique that had been regarded as the gold standard is the semi-automated interactive thresholding technique Cumulus™, which was introduced by Byng et al. and allows separation of mammographic dense from fatty tissue, and thus calculates percentage mammographic density [30]. Cumulus™ has been used in numerous epidemiological studies and demonstrates a higher reproducibility compared to BI-RADS visual assessment [31]. A severe limitation of the interactive thresholding method is that it relies on 2D images and is heavily operator dependent. Meanwhile, 3D mammography-based breast density measurement systems have become available. Highnam et al. [32] and van Engeland et al. [33] have introduced approaches that calculate a quantity, referred to as the thickness of the examined fibroglandular tissue. To date, the Food and Drug Administration has approved Quantra and Volpara for mammography-based volumetric quantitative breast density measurements, which allow volumetric, quantitative measurements of the absolute breast tissue [26, 27, 34–36]. However, it must be noted that breast density calculations based on mammography may also vary due to tissue compression and breast positioning.

### Ultrasound

Ultrasound (US) of the breast is a ubiquitous and cost-effective yet relatively operator-dependent imaging modality, which is easy to perform without the need for intravenous contrast application or ionizing radiation. To date, breast US cannot be reliably used for either a qualitative or quantitative assessment of breast density. However, the latest version of the US BI-RADS atlas recommends a classification of breast tissue composition with US in three descriptive categories: ACR-US-a, homogeneous background echotexture—fat; ACR-US-b, homogeneous background echotexture—fibroglandular; and ACR-US-c, heterogeneous background echotexture [6]. To overcome the drawback of handheld US, automated 3D whole-breast US (ABUS) has been introduced [37] and attempts have been made also to assess breast density with 3D ABUS using semi-automated techniques [38•, 39]. Initial results suggest that ABUS might provide 3D volumetric imaging and accurate breast density measurement [38•, 39]. In asymptomatic women with dense breast US may be a supplemental screening modality adjunct to mammography to enable the detection of additional breast cancers invisible on mammography [40].

### Magnetic Resonance Imaging

Breast density assessment with mammography requires the use of ionizing radiation and compression of the breast, which is often uncomfortable or even painful for the patient. Mammography is a 2D method assessing the breast, which is a 3D structure, and therefore is likely not to provide an accurate absolute, but rather a rough estimate. Additionally, except for R2 Quantra, Volpara, and Densitas, most of the other methods require interaction from radiologists. To address the problems of mammography-based breast density assessment, promising approaches of volumetric, quantitative assessment of the amount of fibroglandular tissue on magnetic resonance imaging (MRI) have been developed and tested.

In contrast to mammography, MRI allows radiation- and compression-free 3D imaging of the breasts and is also able to include breast areas near the chest wall and axilla. MRI provides images related to the fat and water composition of the breast. Since the water composition is highly correlated with the prevalence of fibroglandular tissue, these images can be used for slice-by-slice segmentation of fibroglandular and fatty tissues for quantitative breast density assessment [41]. Many of the currently available approaches rely on the use of T1-weighted sequences, which provide gray-scale images and therefore not enough tissue contrast to allow an objective measurement of breast parenchyma. In addition, most of these approaches require user interaction for breast area segmentation or threshold adjustments [33, 42–47].

To allow precise segmentation, one of the most important steps is the exact definition of breast and tissue borders. The boundaries for the segmentation usually are the anterior border of the major pectoral muscle and the ventral chest wall. As cranial and caudal boundaries, the inferior border of the manubrium sterni and the submammary fold are chosen. For the definition of the lateral borders, Nie et al. [47] proposed an algorithm called the V-shaped cut, which is defined as the area between the lateral border of each major pectoralis muscle and the spinous process at the level of the aortic arch. However, depending on the field of view, the spinous process is not always within the acquired breast volume, and therefore, the V-shaped cut is not always applicable. Moreover, preferentially, the variable subcutaneous fatty tissue of the cleavage should also be excluded from the segmentation. To overcome these, atlas- [48, 49] or template-aided [50] semi-automated approaches with predefined breast models and automated adaption in real time have been explored for individual breast segmentation and the individual outcomes have yielded high accuracy and robustness.

**Fig. 2:**
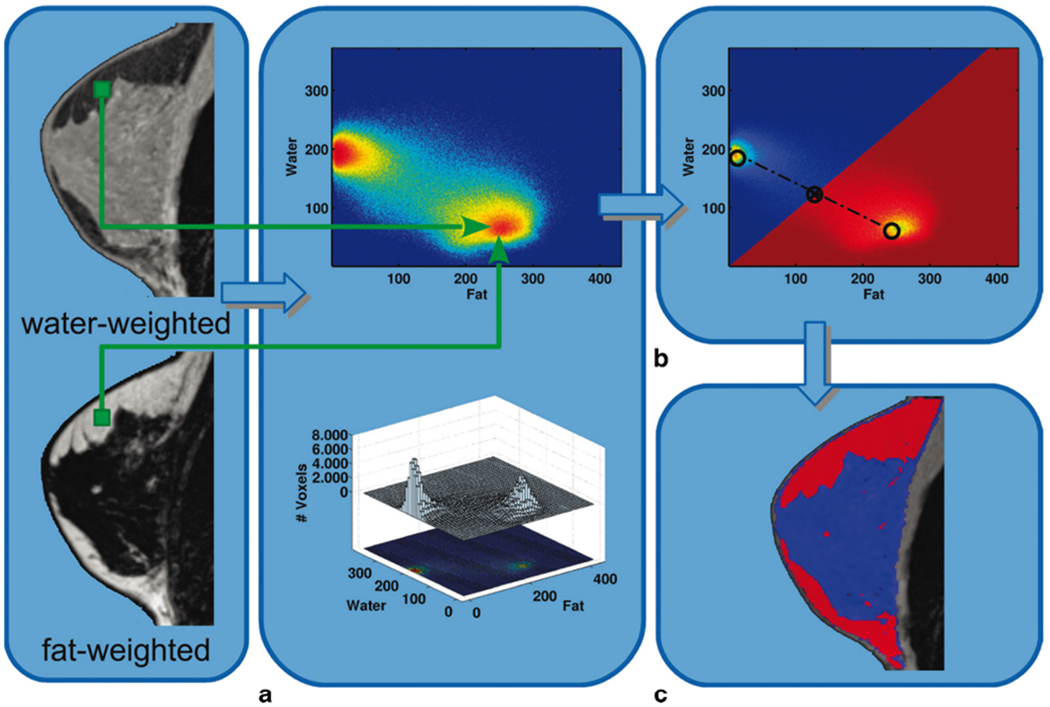
Diagram of the process of fibroglandular tissue segmentation. For each individual breast and water-/fat-based sequence, the program automatically segments an individual breast model, representing the identical 3D breast volume, with the exclusion of the skin and the pectoralis muscle. (A) The signal intensity (SI) values of fat- and water-weighted pixel intensities were recorded and collected into a 2D histogram (top image). At the bottom, there is the 3D illustration of the histogram. (B) Thresholds for the corresponding fat and water SI values were automatically calculated by dividing the histogram into two regions half the distance between the two cluster peaks of the bimodal distribution of measured SI values. (C) Graphical illustration of the assignment for each voxel to be either fat tissue (red) or dense tissue (blue) into the 3D breast model. Published with permission from [50] https://insights.ovid.com/pubmed?pmid=25333307

Besides semi-automated approaches, there are already fully automated, volumetric measurement approaches available to assess the amount of fibroglandular breast tissue on MRI. Gubern-Merida et al. [51] used an expectation-maximization algorithm based on fuzzy *C*-means clustering, and Wu et al. [49] developed a fully automated segmentation approach based on 2D *C*-means clustering. Wengert et al. introduced an iterative segmentation for the separation of the bivariate signal intensity values on Dixon sequences [50] (Fig. 2). The use of Dixon sequences for MRI-based measurements of the amount of fibroglandular tissue has been suggested previously [52] and tested with promising results [50, 53, 54]. Dixon sequences allowed improved reproducibility and accuracy of breast density measurements compared to conventional sequences [55, 56•]. The integration of Dixon sequences into standard clinical MRI protocols for dynamic contrast-enhanced imaging, as well as fibroglandular tissue quantification, is easily possible [55, 57]. Therefore, a recommendation for objective fibroglandular tissue segmentation derived from high-resolution Dixon sequences as the MRI-based reference standard for the assessment of the amount of FGT seems practicable [55, 57–59].

**Fig. 3:**
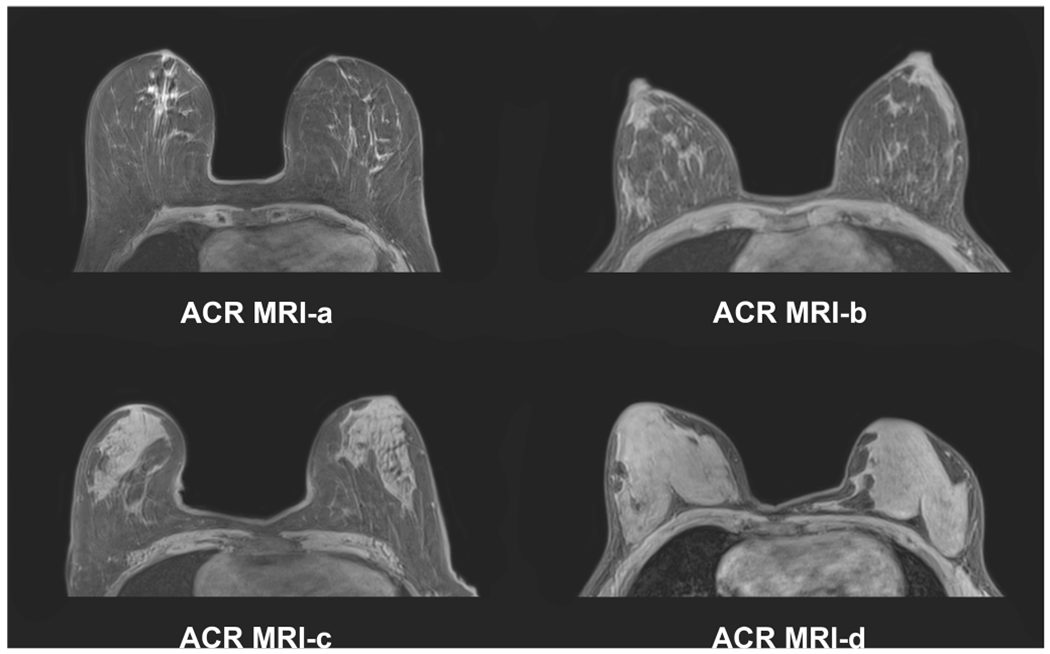
Example images of the four breast density/composition categories defined by the 5th edition of the BI-RADS MRI atlas with four categories similar to mammography. ACR MRI-a, almost entirely fat; ACR MRI-b, scattered fibroglandular tissue; ACR MRI-c, heterogeneous fibroglandular tissue; and ACR MRI-d, extreme fibroglandular tissue

However, it must be pointed out that while fully automated, volumetric MRI-based measurement approaches provide percentage values of breast density, such values are currently not included in the 5th edition of the BI-RADS lexicon. The MRI BI-RADS lexicon currently contains the recommendation to assess the amount of fibroglandular tissue with MRI similar to mammography with four classes (Fig. 3): ACR-MRI-a, almost entirely fat; ACR-MRI-b, scattered fibroglandular tissue; ACR-MRI-c, heterogeneous fibroglandular tissue; and ACR-MRI-d, extreme fibroglandular tissue [6]. Recent studies have shown that this subjective visual estimation of breast density on mammography and the amount of FGT on MRI are prone to error with great inter- and intra-observer variability, which can be improved by reader training [28••, 60, 61]. Similar to mammography, this therefore seems suboptimal compared with objective assessment approaches [26, 27, 28••, 36, 62].

## Breast Density and the Risk for Breast Cancer

Breast density impacts the risk for breast cancer in different ways:

- Breast density can mask breast cancer due to an overlap with normal breast, i.e., the masking effect reduces the sensitivity of mammography screening.
- Breast density is an independent risk factor for breast cancer.

### Masking Effect

There is good evidence that the sensitivity of mammography decreases with increasing breast density. Increasing breast density leads to the effect of overlapping normal breast tissue resulting in coalescent areas of breast parenchyma and obliteration of tissue asymmetries and underlying tumors especially with 2D imaging modalities [6–8]. This masking effect contributes significantly to the reduced sensitivity of screening mammography [7, 8]. Larger breast tumors and advanced stages with lymphatic involvement are seen more often with higher density categories [63–65]. In addition, interval cancers are also increased with studies reporting a 6- to 17-fold increase in the risk of interval cancers for women with higher breast density categories [12, 13, 66].

Most of the evidence on the reduced sensitivity of mammography in dense breasts is from studies that employed screen film mammography (SFM) [5, 6]. With the implementation of full-field digital mammography (FFDM), the sensitivity of screening mammography for cancer detection in women with higher density categories has significantly improved [10, 67]. Data consistently indicate that with the use of FFDM compared with SFM, the masking effect of dense breasts on cancer detection is greatly reduced [10, 67]. In addition, Kerliwoske et al. [68] showed that FFDM compared with SFM also improves the detection of hormone receptor-negative breast cancers (FFDM 78.5% vs SFM 65.8%, sensitivity *P* = .016, in women aged 40–79 years; 95.2% vs 54.9%, sensitivity, *P* = .007, in women aged 40–49 years). These cancers are usually higher grade, carry a poorer prognosis and often manifest as interval cancers, and presumably constitute some proportion of the cancers masked at SFM screening in women with higher density categories.

Recently, digital breast tomosynthesis (DBT) has been implemented in breast imaging. Several large-scale studies worldwide that investigated DBT in the screening setting have demonstrated an increase in cancer detection as well as significant reduction in recall rates compared with FFDM, which is most likely attributable to a decreased masking effect [69–71]. However, the effect of DBT on breast cancer detection related to breast density is not clear yet. In the STORM trial, Ciatto et al. noted that the incremental cancer detection rate beyond that of FFDM in women screened with combined DBT and mammography was similar for dense and non-dense breasts (2.5 per 1000 vs 2.8 per 1000, respectively) [72]. Further studies will be necessary to provide specific insights into the benefits of DBT for reducing the masking effect in women with higher density.

An additional supplemental screening tool in women with higher density is the whole-breast ultrasound (ABUS). Although there is an incremental cancer detection rate with ABUS beyond that of mammography, this comes at the expense of higher false-positive rates with a PPV_3_ reported as low as 3.2% [40, 73–76]. The Institute for Clinical and Economic Review (ICER) report on “The Comparative Clinical Effectiveness and Value of Supplemental Screening Tests Following Negative Mammography in Women with Dense Breast Tissue” [77••] surmised from the results from 15 studies as well as the results of the ACRIN 6666 study [78]. The reported appropriate estimate for the incremental cancer detection rate with supplemental whole-breast screening US is 2 to 3 per 1000, with a markedly low PPV_3_ of 6–7%. Initial results with ABUS screening in women with dense breast are promising. The addition of ABUS to mammography resulted in the detection of 12.3 per 1000 breast cancers, compared to 4.6 per 1000 by mammography alone [79]. However, the true potential of ABUS in this setting still remains to be fully explored.

To date, there is no data available on the benefits of supplemental screening with MRI in women with higher breast density categories. The American Cancer Society (ACS) guidelines neither recommend for nor against screening MRI in women with the risk factor of a higher breast density [80–82]. Screening with MRI is currently reserved for women with a lifetime risk of > 20% [80] and has proven to be superior to both mammography and US for breast cancer detection [83–85]. The Dutch DENSE trial investigates the effectiveness and cost-effectiveness of screening with mammography and MRI compared with those of screening with mammography alone in women with extremely dense breasts [86]. The study has finished enrolment, and the results are eagerly anticipated to better define the role of MRI in this patient population. At this time, the value of supplemental breast MRI screening in women with higher breast density categories remains unclear, and currently, there is no evidence supporting its use.

### Independent Risk Factor

Although the masking effect of breast cancer in dense breast is important, breast density is also an independent risk factor for breast cancer [13, 15, 87, 88]. Breast density refers to epithelial and glandular structures in the breast that are the origin of most breast cancers. It therefore seems logical that an increased breast density elevates the chances of developing breast cancer, i.e., the more epithelial tissue, the greater the chance that cancer may arise in one of the epithelial cells [89].

There is debate as to what extent breast density constitutes an independent risk factor for breast cancer, yet numerous studies have demonstrated an association between breast density and risk for breast cancer that is more than merely a masking bias and cannot be explained by the reduced sensitivity of mammography alone.

In the literature, it has been reported that a linear increase in breast density is associated with a four- to sixfold increase in the risk of breast cancer [31]. In a meta-analysis by McCormack et al. that investigated breast density as an independent risk factor for breast cancer, the relative risk associated with dense breasts was 2.92 for breasts that were 50–74% dense and 4.64 for breasts that were 75% or more dense [13]. Boyd et al. summarized studies that evaluated breast cancer risk with respect to quantitatively measured tissue density, and the odds ratio of the risk for breast cancer ranged from 3.6 to 6.0 [31]. It must be pointed out that the majority of the studies that investigated the associations of breast cancer risk and breast density did not use the ACR BI-RADS categories but quantitative measures or different classification such as the Wolfe classification. It has been demonstrated that the use of the BI-RADS classification results in a similar but milder association of risk with breast density [89].

In many of the studies published on the associations of the relative risk of breast cancer with mammographic density, women with almost entirely fatty breast were compared with women with extremely dense breast. As only approximately 10% of women have either extremely dense or almost entirely fatty breasts, the results are potentially misleading. Nevertheless, when comparing the relative risk for cancer in women with dense breasts to the risk in the average patient, i.e., a tissue density approximately halfway between the two middle categories of scattered areas of fibroglandular density and heterogeneously dense, the relative risk in women with heterogeneously dense breasts compared with the average woman is approximately 1.2 and in women with extremely dense breasts compared with the average woman approximately 2.1.

Although the relative risk of breast density is much smaller than that of other major risk factors for breast cancer, such as age, family history, reproductive history, and genetic mutations, breast density is still a risk factor. Breast density can be easily assessed with mammography, and mammographically dense breasts are relatively common (approximately 50% of the screening population). It therefore stands to reason that the risk factor of breast density alone contributes far more cancer risk to the population than other much stronger risk factors that are less common, such as BRCA mutations or high-risk status [13, 31, 89].

Efforts have also been made to investigate the correlation of breast cancer risk with the amount of fibroglandular tissue on MRI. King et al. investigated the relationships between breast cancer and the amount of fibroglandular tissue and level of background parenchymal enhancement (BPE) on MRI [90]. They found that breast cancer odds ratio increased significantly with increasing BPE. Odds ratio also increased with increasing fibroglandular tissue, but the BPE findings remained significant after adjustment for fibroglandular tissue. Dontchos et al. investigate whether qualitative MRI assessments of BPE, fibroglandular tissue, and mammographic breast density are associated with the risk of developing breast cancer in high-risk women [91]. In this study, greater BPE was associated with a higher probability of developing breast cancer. However, there was no significant association between fibroglandular tissue or mammographic breast density and cancer development (*P* = .5 and *P* = .4). Initial data indicate that with MRI, BPE might a more valuable imaging biomarker for breast cancer risk than fibroglandular tissue. However further studies are warranted to confirm these findings.

## Conclusion

Breast density has become an important topic in breast imaging. Breast density impacts the risk for breast cancer in different ways. On one hand, breast density reduces the sensitivity of mammography due to a masking effect, and on the other hand, there is evidence that breast density is an independent risk factor for breast cancer. Although the extent to which it is an independent risk factor remains controversial, there is a consensus that the increased breast cancer risk is not solely attributable to the masking effect [89]. This emphasizes the potential of breast density for risk prediction and stratification and that breast density might become a valuable tool in determining the best screening plan for each woman and to guide supplemental screening methods. However, to be used in this context, breast density assessment must be reliable, reproducible, and accurate. Breast density has been mainly assessed with mammography using qualitatively subjective visual inspection via the ACR BI-RADS classification. Due to substantial intra-/inter-reader variability, semi-/automated volumetric breast density measurement approaches with both mammography and MRI have been developed with excellent results. Initial attempts for automated volumetric breast density measurements with ABUS are promising. It is expected that these advances in breast density assessment will further define its role in breast cancer risk assessment and help to tailor breast cancer screening strategies to an individual woman’s risk, values, and preferences while also accounting for cost, potential harms, and patient-important outcomes.

## Figures and Tables

**Table 1:** Summary of endogenous and exogenous factors influencing breast tissue composition to increased breast density (does not claim completeness)

Endogenous factors	Exogenous factors
Older age/postmenopause	Smoking
High parity/nulliparity	Alcohol
High body mass index	HRT
Circulating estrogens/IGF-1	Oral contraceptive
African-American	Obesity
Early age at menarche (≤ 12a)	Sedentary time
Older age at first live birth	Physical inactivity
CYP1A2 status	Tamoxifen/vit C, D/folate/NSAID
